# Child health in São Paulo, Brazil: doing things right but with new concerns about anemia and asthma

**DOI:** 10.1590/S1516-31802001000500001

**Published:** 2001-09-01

The metropolitan area of São Paulo is now one of the most densely populated in the world. One century ago, São Paulo was only a small town on the verge of undergoing Brazil's first industrialization. Since then, radical changes have been observed among health indicators. The most impressive change related to child health in the latter part of this period has been reported by scientists from the Department of Nutrition of the University of São Paulo, led by Professor Carlos Monteiro.

Two consecutive household surveys were undertaken in the mid-1980s and mid-1990s in the city of S. Paulo, Brazil. These made it possible to establish time trends for several child health determinants and indicators as well as analyzing the relationships among them. Random samples of the population aged from zero to 59 months were studied: 1,016 children in the period 1984-85 and 1,280 children in 1995-96. The results were published in a special issue of "*Revista de Saúde Pública*" (vol. 34 no. 6, suppl., São Paulo, Dec. 2000, the full text of which is available at www.scielo.br/rsp).

Several good pieces of news were disclosed from these two surveys:

1. Family income doubled and the proportion of low-income families was reduced by 50%, while average maternal schooling increased by 1.5 years and maternal illiteracy was almost eradicated. Income concentration increased during the period.
2. Although the proportion of children living in shantytowns was the same (almost 12%) in both surveys, the characteristics of housing in shantytowns showed an impressive improvement in the period between the surveys, as did the access of this population to water supply and garbage disposal services.
3. There was a significant increase in rooming-in in maternity hospitals, the number of routine visits for babies in their first year of life and, particularly, the universal outreach achieved for DPT, measles and tuberculosis vaccinations. Unfavorable trends were seen regarding the slight and clearly insufficient growth in prenatal care, the still high percentage of cesarean sections (almost 50%), and the limited routine visits for children after their first year of age.
4. The birth weight distribution did not change substantially across the study period. However, there is evidence of changes when different socioeconomic strata are considered separately. Among the lower strata, the trends were positive and this seems to have been due to increases in intrauterine growth as a result of an improvement in family purchasing power, women's weight and height, prenatal care and, possibly, the reduction in smoking. Among the higher socioeconomic strata, birth weight trends have been negative, apparently due to an increase in premature births of unknown origin.
5. Child malnutrition came under control in the city and became relatively rare, even among the poorest families. The risk of obesity remained low and restricted to the richest families. Positive changes in distal (family income and maternal schooling) and intermediate (sanitation, access to heath services and previous reproductive history) determinants of child nutritional status substantially explained part of the decline in the prevalence of malnutrition seen from the mid-1980s to mid-1990s.
6. Over the time span from the first to the second survey, there was a substantial reduction in the prevalence of all parasites (from 30.9% to 10.7%), helminthes in general (from 22.3% to 4.8%), giardiasis (from 14.5% to 5.5%) and two or more species of parasites (from 13.1% to 0.5%). A significant decline in prevalence was observed in all social strata and the inverse association between income and intestinal parasites remained unchanged in the period. Positive changes in distal (family income and maternal schooling) and intermediate (housing, sanitation, and access to health care) determinants of helminthic disease could substantially explain part of its decline over the period. The decline in giardiasis was attributed to improvement in maternal schooling, housing and sanitation. The doubling of the rate of attendance at day care nurseries may have restricted the rate of decline in giardiasis prevalence during the study period.
7. Over the time span from the first to the second survey, there was a substantial reduction in both the prevalence of diarrhea (from 1.70% to 0.90%) and the hospitalizations due to the disease (from 2.21 to 0.79 hospitalizations per 100 children-year). The most significant reduction was observed within the third poorest stratum of families, thereby narrowing the social gradient relating to the disease. The increase in family income and improvement in water supply could substantially explain part of the decline in the disease and, for children under two years of age, a discrete increase in breast-feeding may have also played a positive role.

In contrast, new challenges for child health planners were detected, such as:

1. Over the time span from the first to the second survey, there was a significant reduction in the average hemoglobin concentration (from 11.6 g/dl to 11.0 g/dl), as well as a considerable increase in anemia prevalence (from 35.6% to 46.9%). Unfavorable trends were observed in both sexes, all age groups and all income strata. Trends were still less favorable among the poorest families, aggravating the social burden related to child anemia. Changes in distal (family income and maternal schooling) and proximal (breast or bottle-feeding) determinants of child anemia were positive over the study period and therefore they cannot explain the increase in the disease. A low iron diet could explain the high prevalence of anemia in both surveys but could not explain its further increase.
2. Over the time span from the first to the second survey, there was a substantial increase in the prevalence of both upper (from 22.2% to 38.8%) and lower respiratory diseases (from 6.0% to 10.0% and from 0.8% to 2.8%, without and with wheezing, respectively). In the case of upper respiratory disease and lower respiratory disease without wheezing, an increase in prevalence was observed across all social strata, which did not interfere with the slightly less favorable situation of the lower income groups. In the case of lower respiratory disease with wheezing, the increase was only observed among middle and low-income groups, being particularly high among the lower income group, yielding a significant inverse gradient between income and respiratory disease. Positive changes in distal (family income and maternal schooling) and intermediate determinants related to housing characteristics would have resulted in a decline, not an increase, in the prevalence of respiratory diseases in the city. The doubling of the rate of attendance at day care nurseries seen over the period could have counterbalanced the positive effect due to socioeconomic and housing variables but would not be enough to explain an increase in the disease.

Concluding, these results are very important for establishing new targets for child health. First, the improvement of prenatal and delivery care is the most important issue facing the National Health System in São Paulo. To achieve this improvement, less money will need to be spent than is now spent on dialysis and transplantation, which both have very influential lobby groups. Second, the reason for the increased anemia and wheezing between the two survey periods is not clear. Although several explanations can be found by reviewing the medical literature, there is an absolute need for new studies focusing on these new challenges, especially aspects of respiratory diseases.

